# Surgeon-General Duncan Alexander Campbell Fraser

**Published:** 1912-09-14

**Authors:** 


Surgeon-General Duncan Alexander Campbell Fraser has died at Cheltenham at the age of eighty. Afer taking his medical degree at Edinburgh in 1853 he served for nearly forty years in the Army Medical Service, his Staff appointments including the medical commands at Netley and Malta. He held the Star of Roumania for services as a Commissioner of the Red Cross Society in the Russo-Turkish war.

